# Predictors of Hypertension in a Population of Undergraduate Students in Sierra Leone

**DOI:** 10.1155/2017/8196362

**Published:** 2017-08-03

**Authors:** Aiah Lebbie, Richard Wadsworth, Janette Saidu, Camilla Bangura

**Affiliations:** ^1^Department of Biological Sciences, Njala University, PMB, Freetown, Sierra Leone; ^2^Institute of Food Technology, Nutrition & Consumer Studies, Njala University, PMB, Freetown, Sierra Leone

## Abstract

We report on the first survey of hypertension in undergraduates in Sierra Leone. Levels of hypertension (12%) and obesity (4%) appear low compared to the general population but given the rapid increase of both and the expectation that many graduates will enter the formal employment sector and a sedentary lifestyle, there is still cause for concern. We measured their BMI (body mass index) and used a questionnaire to investigate demographic and lifestyle choices. In agreement with most authorities, we found that BMI and age were statistically significant predictors of systolic and diastolic blood pressure but that the explanatory power was low (*r* = 0.21 to 0.27). Men may be more sensitive than women to an increase in BMI on blood pressure (*p* < 0.1). We failed to find statistically significant relationships with ethnicity, religion, stress, course of study, levels of physical activity, diet, smoking, or consumption of caffeine and alcohol. Family history of hypertension, consumption of red palm oil, and self-diagnosed attacks of typhoid fever were close to conventional levels of significance (*p* < 0.1). We intend to use this as a baseline for longitudinal studies to assess risks and suggest appropriate public health action.

## 1. Introduction

Most investigations into public health in Sierra Leone have concentrated on “exciting” diseases, such as Ebola virus disease (EVD), and on acute problems associated with perinatal and maternal mortality; these continue to receive national and international attention. Research into noncommunicable chronic health problems such as hypertension and obesity has been limited, and we have found only four papers in the PubMed database [1–4]; in addition there is the WHO [5] country profile. From these papers, the rate of adult hypertension is somewhere from a low of 15% [2] to over 44% [4] with the WHO [5], settling on a prevalence of adult (over 25 years) hypertension of 36.7% and 36.0%, for men and women, respectively. This is higher than the nearby country of Ghana (28.3% and 32.8% for urban and rural populations) [6, 7] or the year 2000 survey of Sub-Saharan Africa (SSA) (28.3% and 26.9% for men and women) [8], but lower than rates in rural southeast Nigeria (50.2% and 44.8% for men and women) [9]. Recent levels in Nigeria are much higher than ten years previously [10, 11] when levels of only 10% and 11.2% were recorded. Comparison of rates is hampered by different methodologies and selection biases. Where the sample size is large and methodology is consistent, others have shown that rates of hypertension can increase rapidly, for example, in China from 18% in 2002 to 33.6% in 2010 [12].

Of the four papers on blood pressure (BP) in Sierra Leone, recorded in PubMed, two of the papers [1, 2] utilize data collected during the civil war and may reflect a population under a variety of severe psychological and physiological stresses and one paper [3] relates to patients attending a medical clinic. Lisk and others [2] found a significant relation with age and gender but not rural or urban location. Other researchers [1, 3] only found age to be significant, and data collected on demographic, dietary, and social factors, including smoking and consumption of alcohol, salt, palm oil, and kola nuts were not statistically significant predictors [2]. In a cross-sectional study conducted in the Gambia and Sierra Leone, over 40% prevalence rates of hypertension were recorded in both male and female patients [4].

West Africa is noted as a location where many people have high blood pressure; anecdotally hypertension is a major cause of early death, especially among men, but few autopsies are performed and national statistics are of variable quality. In Sierra Leone, no studies have examined the prevalence of and associated risks of hypertension among young university students, despite the evidence that hypertension is a risk for undergraduates in other countries in the region [13]. Given the asymptomatic nature of hypertension, there is a need to determine populations that would be most vulnerable and to identify appropriate risk factors to provide the needed public health support. One of the problems with studying hypertension is the large number of factors that can influence blood pressure. With this in mind, we measured and interviewed 345 undergraduates on the main campus of Njala University to try and determine what physical and environmental features best predicted systolic blood pressure (BP-S) and diastolic blood pressure (BP-D) and whether there are public health implications or recommendations that needed to be made.

## 2. Materials and Methods

### 2.1. Study Site and Approach

The study was conducted on the main campus of Njala University, located in a rural setting in the Moyamba District of Southern Sierra Leone, and attracts students from across the country primarily interested in pursuing university degrees in agriculture, science, and technology. Data collection began in March and ended in May 2016 and was intended to establish a baseline data for a long-term longitudinal study with the students.

### 2.2. Blood Pressure and Anthropometric Measurements

Blood pressure and anthropometric measurements were done with the assistance of one trained nurse and 3 student volunteers. Measurements for blood pressure, height, and weight, as well as circumferences for arm, chest/bust, hip, and waist, were made. A digital sphygmomanometer (OMROM Blood Pressure Monitor, Model: BP765) with two different cuff sizes was used to record the conventional blood pressure (systolic and diastolic) from the distal part of the left arm of the students after resting for at least 10 minutes in a resting position [14]. The average of two BP measurements of between 5 and 6 minutes' interval was taken and recorded. This equipment consistently gave the same readings when used on different sets of people on different occasions before we commenced actual BP recording of the students. Participants' weights were measured on a manual scale to one decimal figure in kilograms, and they were encouraged to put on light clothing and put aside phones and step out of their shoes before the measurement was done. A marked “height wall” was used to measure the heights in the nearest centimetre, while a nonelastic tape measured the circumferences of the arm, chest/bust, waist, and hip.

### 2.3. Questionnaire and Sociodemographic Characteristics

A questionnaire divided into four sections covering basic sociodemographic information, food recall data, personal and family health, and physical exercise and lifestyle was given to each study participant to fill out. Upon completion of the questionnaire, each participant was given the equivalent amount of $1 (Le 5,000) to buy telephone credit to call the PI should they have any questions or desire to opt out of the study.

### 2.4. Data Analyses and Statistical Approach

Data from the questionnaires was recorded onto an Excel spreadsheet primarily as a mixture of ordinal and categorical data (e.g., gender, preferred type of alcohol) with some continuous variables (e.g., frequency of playing sports) categorised into a limited number of choices (e.g., “every day,” “3-4 times per week,” “once a week,” “occasionally,” etc.). Anthropometric measurements (e.g., body mass index (BMI)) are treated as continuous variables.

The simplest relevant statistical approach was adopted for each analysis. Statistical significance was mainly tested using ANOVA (analysis of variance) and linear regression with some use of mixed models (e.g., using BMI, age, and gender to predict BP). Quantile and median regression gave similar results to linear regression using maximum likelihood and are not therefore reported. Sample size (and degrees of freedom) varies slightly with the different variables being analysed as not all respondents answered all questions in a usable manner.

### 2.5. Ethical Approval Process

In February 2016, ethical approval to conduct the study was sought and obtained from the Njala University Institutional Review Board. We first publicized the study on the Njala campus, and students were encouraged to participate in a campus-wide briefing on the study objectives and data collection process in the university auditorium. A question and answer session was followed by information sharing on ethical approval and consent process before data collection commenced. Interested students were given a prepared consent form to take home and read; 345 (82.7%) completed forms were returned (after collecting and cleaning the data, 332 usable responses were obtained). The consent forms were countersigned and witnessed by the first and third authors. Students who agreed to participate had their BP measured and then had their anthropometric measurements recorded and completed a “lifestyle” and sociodemographic questionnaire.

## 3. Results

### 3.1. General Characteristics of the Population

The general characteristics of the sample are shown in Table 1 for selected demographic and biological attributes. More male students participated in the study than female students, with the median age of males being 25 years (quartile 22–27) and that of the female students being 23 years (quartile 21–25). Male students were generally taller than female students, and median BMI for male students was 2.1 kg/m^2^ less than that of female students (Table 1). The percentage of obesity was higher among female students than male students, with the number of siblings higher for male than female students. Religious beliefs and practices were more engendered, with female students likely to be Christian than Muslim. Most of the students were single, with slightly more married female than male students.

### 3.2. Risk Factors and Blood Pressure

Factors that were significantly predictors of either BP-S or BP-D and factors that are close to conventional levels of statistical significance (*p* < 0.1) are shown in Table 2. Factors that were not significant are listed briefly in Table 3.

#### 3.2.1. Body Morphology

We measured the weight, height, waist, and hip circumferences of each respondent. Median BMI is 21.7 (interquartile range 20.1 to 23.8). The majority (251, 76%) were in the “normal” range (BMI of 18.5 to 25), 18 were “underweight” and 46 “overweight”, 14 were “obese,” and only one individual was categorised as “extremely obese” (with a BMI greater than 40). Women were on average 2.1 BMI units larger than the men (median values 23.4 versus 21.3) and this was statistically significant (one-way ANOVA, *p* < 0.001, *F* = 26.0, *df* = 328).

**Figure 1:**
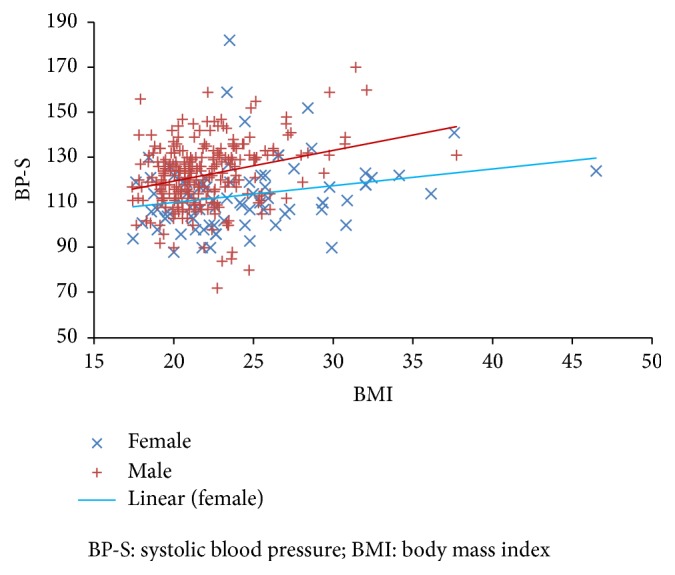
Systolic blood pressure (BP-S) and BMI for men and women.

**Figure 2:**
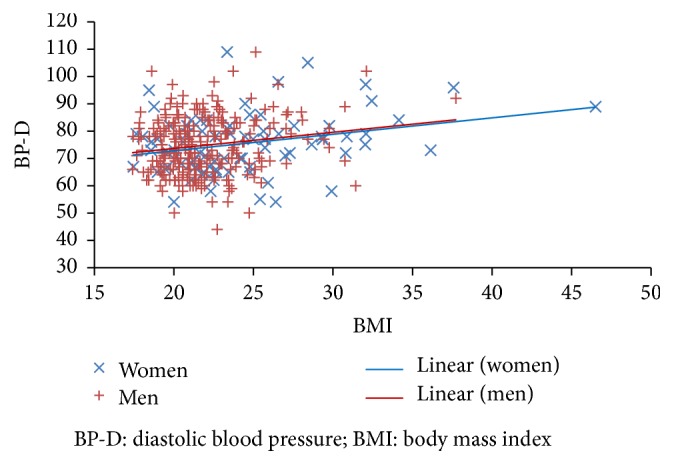
Diastolic blood pressure (BP-D) and BMI for men and women.

BMI is a significant predictor of BP-S and BP-D but the relationships for BP-S ((1) and (3)) are different for men and women, with an increase in BMI having a greater effect on BP-S for men than for women (see Figure 1); the relationship for BP-D ((2) and (4)) is virtually the same for both genders (Figure 2). Unfortunately, while being statistically significant, linear regression has only a weak explanatory capability with adjusted *r*^2^ values of less than 10%.

Men:
  (1) BP-S=91.7+1.38∗BMIadjusted  r2=0.072,  F=20.4,  df=251,  p<0.001
  (2) BP-D=61.5+0.60∗BMIadjusted  r2=0.023,  F=7.11,  df=251,  p<0.01.
Women:
  (3) BP-S=95.5+0.73∗BMIadjusted  r2=0.044,  F=4.56,  df=77,  p<0.05
  (4) BP-D=61.3+0.58∗BMIadjusted  r2=0.062,  F=6.09,  df=77,  p<0.05.

The difference in slope in (1) and (3) is not quite statistically significant (*p* = 0.078, *t* = 1.768, and *df* = 341). The difference between (2) and (4) (for BP-D) is not significant. Adding the variable “age” (of student) into the equations does not improve either the statistical significance or explanatory power of the equations for BP-S, but it does slightly in a combined equation for BP-D for men and women (5).

Men and women:
  (5) BP-D=56.5+0.434∗BMI+0.344∗Ageadjusted  r2=0.062,  F=11.9,  p<0.001,  df=329.

#### 3.2.2. Food Intake Recall

One feature of West African cuisine is a heavy reliance on red palm oil, and this is considered one of the least healthy vegetable oils but is consumed by most of our respondents on a daily or weekly basis (Table 2). Frequency of consumption of palm oil was not significant at a conventional level of 0.05 but was positively related to increased BP-S (*p* = 0.068). We could not measure actual consumption, only approximate frequency (“every day,” “3 to 4 times per week,” “once per week,” “occasionally,” and “never”). Consumption of other vegetable oils (used for frying foods) does not appear related to BP (or BMI). Sierra Leone produces many types of leafy green vegetables and we enquired as to peoples favourite foods, taboo foods, allergies, and consumption of vegetables (which was generally interpreted by our respondents as being “western” foods such as lettuce, carrots, and cabbage and relatively few classed okra, garden eggs, or any of the “leafy green vegetables” as vegetables). By far the favourite meal of our sample is cassava leaf sauce (62%, 215) (although 22 people reported being allergic to some forms of cassava). There was no statistically significant relationship between favourite foods, taboo foods, allergies, and consumption of vegetables and BP, although consumption of tomatoes was significant (*p* = 0.034) (Table 2).

#### 3.2.3. Lifestyle and General Health

At Njala, only 17 respondents (5%) admitted smoking and this is much lower than the 43% (male) and 11% (female) levels given by the WHO [15] for Sierra Leone adults. Contrary to expectation, there was no statistical difference in BP. Alcohol consumption seems relatively low by European standards and those that consumed alcohol had a slightly lower BP-D than nonconsumers but this was not significant at conventional levels.

Most undergraduates consider themselves to be healthy (303 consider they are “very” or “moderately” heathy); however, many (42%) have been affected by malaria since coming to Njala campus. Reported attacks of malaria had no effect on BP-S; however, those reporting an attack of typhoid (often as “typhoid-malaria”) had lower median BP-S (117 versus 126) but this is not quite significant at the 0.05 level (*p* = 0.08, one-way ANOVA, *F* = 3.04, and *df* = 332), and reports of other illnesses had no statistical significance. Family health in terms of parents and grandparents diagnosed with diabetes, heart attacks, and obesity had no statistical effect on BP with the exception of a recent death from “high blood pressure.” Married respondents had slightly higher BP-D than singles but this was not quite significant at conventional levels (*p* = 0.093).

### 3.3. Factors with No Significant Relation to Blood Pressure

Table 3 lists those factors that were tested and where the “*p*” values were always greater than 0.1. We performed one-way ANOVA on many sociodemographic and lifestyle attributes to determine their possible impact on blood pressure including the following:

- place of origin (northern, southern, or eastern province and western area) in Sierra Lone,
- ethnicity (tribe) of father and mother separately, and for a subset where the tribe of both parents was the same,
- number of siblings and birth order,
- religion (males split almost equally between Islam and Christianity (125 : 119); women were more likely to be Christian (50–30); only three “preferred not to answer”).

There were no statistically significant relationships with place of origin or ethnicity with BP. We had hypothesised that ethnicity (as a surrogate for genetics) and place of birth (as a surrogate for environment, e.g., coastal versus inland) might be factors affecting either early diet (e.g., relative availability of fish on the coast or meat in the interior) or levels of activity (e.g., plains versus mountains) and hence BP.

We asked about the spacing of siblings (number of years between them and siblings) but the quality of responses prevented detailed analysis. Large family size and a short period between siblings (less than 18 months) are known to have an adverse effect on some aspects of health and mortality, but we found no statistically significant effect on blood pressure (possibly because less healthy siblings are less likely to do well enough in school to get to university?).

Periodic fasting, a feature for observant Muslims might affect metabolism, but we found no effect on mean BP. An* F*-test showed significantly more variance in the Christian population than in the Muslim population for both men and women (women *F* = 2.45, *p* < 0.01, *df* = 49, 29; men *F* = 2.04, *p* < 0.001, *df* = 121, 125); we are uncertain why this might be the case.

In our sample, less than one-third of undergraduates (31.4%) reported drinking alcohol, and for the purpose of analysis alcohol was grouped into “beers and wine,” “spirits,” and “palm wine” and consumption as “every day,” “3 to 4 times per week,” and so forth. Consumption of stimulants was categorised into “energy drinks,” “coffee, kola nuts, and coca cola,” and noncaffeinated “soft drinks” (mostly locally produced ginger beer and synthetic apple juice). Consumption of energy drinks was very widespread (83% of male and 70% of female students). There was no significant relationship (one-way ANOVA) with alcohol or caffeine (BP for men was higher for those that took energy drinks, but lower for women; neither relationship was close to significance with *p* > 0.1).

Levels of physical activity were assessed through frequency of participating in organised sports (football and volley ball). Male students at Njala reported much more sporting activity than female students and had lower BMI on average than nonsports players, but this was not sufficient to result in a significantly lower BP.

Anecdotally some university courses are harder than others, and we hypothesised that there would be a change in the levels of stress as the undergraduates passed through the four years of the degree course or whether certain schools (e.g., agriculture, education, technology, and environmental sciences) were inherently more stressful than others. One-way ANOVA revealed no significant differences between the seven schools represented by the male respondents and the four schools represented by the female students.

Students reported a wide range of stresses relating to student life, such as poor accommodation (excessive noise, limited water supply), exams and essays, money, and relationships. These stresses on their own and in aggregate did not statistically affect BP. One symptom of stress can be problems sleeping, and a large proportion (151, 44%) reported only sleeping 5 hours per night (and just 6% reported sleeping 8 hours per night). Lack of sleep was not a predictor of BP. Ulcers can also be related to stress and 30 (8.6%) reported they had ulcers (although whether this has been formally diagnosed is unknown). Ulcers might lead to a reduction in consumption of oil and chili pepper, but this was not detectable in our data.

## 4. Discussion

Values of BMI (body mass index) in the student population are broadly consistent with estimates for adults made by the WHO between 1980 and 2009, which shows a monotonic increase from 21.4 to 24.1 for women (median 23.4 in our sample) and from 21.4 to 22.7 for men [5] (median 21.3 in our sample). The WHO data does not indicate any interruption of the increase in BMI during the Sierra Leone civil war, but a slight increase in slope after the end of the war in 2001. The BMI of male students was remarkably consistent by year of study. For the women (a smaller sample) there was a significant difference between years, due to a high average value for first-year students (who constituted 56% of the female sample).

In our study population, BMI was a significant predictor of both diastolic and systolic blood pressure, though the increase in BMI had a greater effect on systolic pressure for men than women, and when age was included together with BMI for a combined male and female diastolic pressure, the difference was statistically significant. Body mass index is reported to be positively and independently associated with morbidity and mortality from hypertension, cardiovascular disease, type II diabetes mellitus, and other chronic diseases [16]. In Caucasian populations, a strong association has been depicted between BMI and mortality [17], and positive relationship between BMI and BP has also been reported among African and Asian populations [18–21]. In West Africa, a meta-analysis conducted on 55 articles from 5 countries includes obesity, age, and gender as significant predictors [22] and is similar to studies of adults in Sierra Leone [2, 3]. Significant relationships have also been reported between age and BMI among adolescents in northeast Nigeria [23, 24], in which BMI was found to be a significant factor in predicting hypertension in rural Nigeria. In a small sample (60 students) of obese Nigerian students, Ibhazehiebo and others [25] found a strong, significant correlation (*r* = 0.60) between BMI and blood pressure. Others have also observed statistically significant relationship between BMI and blood pressure in undergraduates in three Nigerian universities [26, 27].

In Europe, the waist-to-hip ratio is sometimes used as an alternative to the BMI to assess risk. Within our dataset, the waist-to-hip ratio was not significantly correlated with BP, possibly due to different patterns of where fat accumulates. The waist-to-height ratio was statistically significant but resulted in regression equations with a lower *r*^2^ than BMI; however, it requires one less piece of equipment to measure it (hip-to-height ratio was not significant). Studies done on populations in Haiti and Benin found the waist-to-height ratio to be a significant predictor of cardiovascular risk [28]; unfortunately, they did not report if this was as effective a predictor as BMI.

Although high blood pressure is common in SSA, a study from Brazil [29] supports a view that it is socioeconomic factors (such as low levels of education and income) and not genetic composition (African, European, or Native American) that are the important predictors. No significant difference between adults in Sierra Leone and the Gambia was found [4], and a meta-analysis also failed to find a consistent relationship with ethnicity [22].

Diet is often considered as a predictor of health; however, comparisons of BP for a small sample of vegetarians (who were all Seventh Day Adventists) with nonvegetarians in Nigeria found no difference [30]. There are very few vegetarians in Sierra Leone; however, consumption of meat is low and the majority of animal protein is provided in the form of marine fish. As Njala University has demonstration units for pigs, chickens, goats, and cattle, meat consumption might be above the national average among the university students. In rural Nigeria [24], a liking for fried chicken was found to be a significant risk factor for high blood pressure among females. In school children in the Democratic Republic of Congo boys with chronic malnutrition had significant higher blood pressure levels, but only low socioeconomic status was identified as a significant risk factor of arterial hypertension [31]. In our study group, the frequency of consumption of palm oil was high and, while being not significant, was positively related to increased systolic blood pressure (*p* = 0.068).

The nicotine in tobacco is capable of increasing blood pressure in otherwise healthy individuals [32], and studies of smoking among West African students reported that smoking is low in a sample of 1,800 Nigerian students, as only 5.7% smoked (7.7% in males and 2.0% in females), and unfortunately, few of them (32%) believed they were incurring any health risk and hence were willing to consider giving up [33]. At Njala University, approximately 5% of the students admitted smoking, a much lower frequency than the 43% (male) and 11% (female) levels given by the WHO [15] for adults in Sierra Leone, but differences in BP are not statistically significant. In a small sample of undergraduate students at Benin University in Nigeria, blood pressure measurements results were similar to studies of Black Americans [34], despite the observation that levels of smoking and alcohol intake among the Nigerian students were lower. While alcohol consumption has been found to be significantly related to higher blood pressure [22], no significant relationship was found in our study population, although men who drank energy drinks had a higher BP than women.

Male students showed more involvement in physical activities such as sports than their female colleagues. Watching of movies (mostly “Nigerian Soap Opera”) was more common among the female students who also showed less preference for sports that were physically challenging. Studies done in South Africa found that male South African students were more physically active than the females but had poorer eating habits [35]. They, however, worry that hypertension is becoming an increasing problem and health problems were starting earlier than expected.

Our study population reported a wide range of stressful experiences, including money, exams and essays, noise, health of family members and themselves, water supply, and relationships. By far, stress related to lack of money is more prevalent among male and female students, given that most of them have to finance their own education, feeding, transportation for getting around the campus, and paying for educational materials and medical treatment when they are sick. While we could not find any significant relationship between any of the observed stress categories and blood pressure, others [36] observed a statistically significant relationship between blood pressure and women with premenstrual syndrome. A recent study has also observed that emotional stress is often ignored as a cause of high blood pressure in SSA [37]. In a behavioural study of under- and postgraduates in 208 Lagos students (equally split male : female, mean age 20.2), Agbu [38] found that the female students were more motivated and felt under greater pressure to get things done and find a job.

## 5. Conclusions

The standard of living in Sub-Sahara Africa (SSA) is on the rise, and families are taking on lifestyles of the developed world with attendant health implications, including weight gain/obesity and hypertension, arising from a higher calorie intake coupled with less physical activity [39, 40]. The magnitude of these changes in most countries across the region is not clear as there are thoughts to be high levels of underdiagnoses and reporting. Odili and others [41] using the “International Database on Home Blood Pressure in Relation to Cardiovascular Outcome” (IDHOCO) consider that hypertension is both underdiagnosed and a major cause of death in SSA. The extent of “masked” hypertension in SSA is described as being “alarming” [42] and a “silent killer” [13].

Levels of hypertension are high in the Sierra Leone adult population and appear to have increased over the last few decades; but the student population at Njala is currently quite healthy with 12% having either high BP-S or BP-D and only 7 (2.1%) having both high BP-S and BP-D. Levels of obesity are low (4%) but likely to increase as they age and with the expectation that they will enter the formal employment sector and a sedentary lifestyle.

Like most other studies, we found a statistically significant correlation between BMI and BP. Work done in South Africa suggests that the critical “cut-off” points for BMI in relation to public health advice on the risks of high blood pressure should be considerably lower than those currently used [43]. Gender and BMI are significant predictors of BP-S. For the combined BP-D equation age is also a significant predictor, but we noted that the age range of our sample is small (interquartile range 22 to 27 years). There is some indication that men are more sensitive to increases in BMI than women (but the difference in slopes of the linear regression equations is not significant at conventional levels, *p* = 0.08).

Consumption of palm oil (increase BP) and recent attacks of “typhoid” fever (decrease BP) are close to conventional levels of statistical significance (*p* < 0.1). Red palm oil is known to be less healthy than some other vegetable oils. In laboratory experiments involving rats, repeatedly heated palm oil was reported to have caused elevation in BP [44]. We have not been able to trace any existing literature on typhoid fever that ties in any relationship with BP, and the self-reported “typhoid fever” by students did not accompany any proof that they had typhoid fever or “malaria fever” (as people in Sierra Leone generally use both terms synonymously until proven otherwise). However, we can only speculate that malaria fever which affects normal blood cell counts might impact on BP. In fact, it has been shown that there is a decrease in blood pressure with malaria attacks [45].

We are tempted to speculate that fasting among the Muslim students might reset their metabolic rates over that of the Christian students, hence the variance in BP. Restrictions on caloric intake have been shown to have favourable outcome for blood pressure and other health outcomes [46]. We were unable to ask about strict adherence to religious tenets to ascertain whether reported religious affiliations of the students actually translated into practices that might influence metabolic rates and hence health-related outcomes. This issue will be included in our long-term studies with the students and develop hypotheses for testing it.

Levels of alcohol consumption and smoking (if honestly reported) are low with less than one-third drinking and just 5% smoking, neither is a statistically significant predictor of BP in our sample. Physical activity slightly reduces BMI but does not significantly improve BP. Students report many sources of stress but these do not significantly increase BP. We did not identify ethnicity, religion, place of birth, or a family history of ill-health as significant predictors of BP.

The Njala campus is a relatively calm and peaceful rural environment; the University has some departments located in the cities of Bo and Freetown, and we would like to conduct a similar survey to see whether the urban environment has a measurable effect on BP. Our sample generally comes from better off sections of society; it would be interesting to conduct similar surveys in more typical communities. We expect many of our undergraduates to enter the formal economy and probably adopt a more sedentary existence thereby increasing their risk factors; whether they can be influenced now before they start to spread out is something we must address.

As the levels of smoking at 5% are much lower than the national statistics (40% for men), it would be interesting to follow this up and see whether students have been influenced by public health messages. This is especially important as relying on conventional medical treatment for chronic conditions like hypertension once someone is affected is unlikely to be effective; in Ghana 93% of patients did not take their prescribed medication, most (96%) stating that the medicines were unaffordable [47].

## Figures and Tables

**Table 1:** Selected characteristics of the student population on Njala campus.

Characteristic	Male		Female	
	Median or percentage	Quartiles	Median or percentage	Quartiles
Number of respondents	252		80	
Age (years)	25	22 : 27	23	21 : 25
Height (cm)	172	167 : 176	161	155 : 165
Weight (kg)	62.5	58 : 69	61.0	53.0 : 70.3
BMI (kg/m^2^)	21.3	20.0 : 23.3	23.4	21.1 : 26.7
Obese (BMI > 30)	2.0%		4.0%	
Number of siblings	4	3 : 6	3	2 : 4
Christian	49.4%		62.5%	
Muslim	50.6%		37.5%	
Married	6.4%		7.5%	

BMI: body mass index.

**Table 2:** Potential risk factors for the student population on Njala campus (probabilities from single factor ANOVA or linear regression).

Attribute	Number	Median BP-S (interquartile)	Significance	Median BP-D (interquartile)	Significance
*Sociodemographic*					
Sex					
Male	250	122 (112 : 131)	*p* < 0.001	75 (67 : 82)	NS
Female	80	112 (103 : 121)		76 (68 : 82)	
Age range					
Age < 25 years	171	118 (108 : 130)	*p* = 0.026	72 (65 : 80)	*p* < 0.001
Age ≥ 25 years	159	121 (111 : 130)		77 (70 : 83)	
*Biological variables*					
BMI					
Obese (I & II)	15	123 (118 : 137)	Regression	84 (74 : 91)	*p* = 0.017
Overweight	46	124 (111 : 132)	*p* = 0.003	77 (70 : 84)	
Normal	251	119 (109 : 128)		73 (67 : 81)	
*Marital status*					
Married	22	124 (115 : 130)	NS	77 (72 : 87)	NS
Single	305	120 (109 : 129)		74 (67 : 82)	(*p* = 0.093)
*Health behaviour*					
Alcoholic intake					
Drink alcohol	109	120 (111 : 129)	NS	72 (66 : 80)	NS
No alcohol	238	119 (109 : 130)		76 (68 : 82)	(*p* = 0.089)
Palm oil intake					
Palm oil > 4 d/w	158	123 (110 : 131)	Regression	75 (67 : 83)	NS
Palm oil 3-4 d/w	153	118 (110 : 128)	*p* = 0.073	73 (67 : 81)	
Palm oil 1-2 d/w	33	115 (108 : 128)		76 (67 : 82)	
Palm oil, never	3	123 (122 : 129)		87 (83 : 87)	
Vegetable intake					
Fresh tomato	83	120 (111 : 130)	NS	79 (69 : 84)	*p* = 0.034
No fresh tomato	263	119 (109 : 129)		73 (67 : 81)	
Self-reported					
Typhoid					
Typhoid recently	38	112 (102 : 122)	*p* = 0.020	69 (65 : 78)	*p* = 0.011
No typhoid	308	120 (110 : 130)		76 (68 : 82)	
Self-reported					
Malaria					
Malaria recently	147	118 (109 : 129)	NS	73 (66 : 81)	NS
No malaria	199	120 (110 : 130)		76 (68 : 83)	(*p* = 0.097)

BP-S: systolic blood pressure; BP-D: diastolic blood pressure; NS: not significant; d/w = days per week.

**Table 3:** Risk Factors with no conventionally statistically significant relations to blood pressure in our sample.

Factor	Categories
Religion	Christian, Muslim, “prefer not to say”
Marital status	Married, single
Place of birth	Northern, southern, eastern province, western area
Place of birth	14 districts
Ethnicity of mother, father	Fula, Koranko, Limba, Mandingo, Mende, Susu, Temne, others
Number of siblings	Only child, 1, 2, 3, 4, 5, 6, 7, 8, 9, more than 9
Birth order	1 (first) through 9 (ninth)
Years to nearest sibling	Insufficient quality data for analysis
School of study	Agriculture, education, environment, health, natural resources, physics/technology, social sciences
Year of study	1st, 2nd, 3rd, or 4th
Physical activity	Play sports (football, volleyball, running), do not play sports
Watch TV	More than 4 days/week, 3-4 days/week, 1-2 days/week, occasionally
Smoker	Yes, no
Drink alcohol	Yes (spirits, wine, beer, palm wine), no
High caffeine intake	Yes (energy drinks, caffeinated drinks, cola nuts), no
Favourite food	Cassava leaf, other leafy green vegetables, beans, “soup,” other
Food allergies and taboos	Cassava, pumpkin, lizard, pig, other
Fresh fruit consumption	More than 4 days/week, 3-4 days/week, 1-2 days/week, occasionally
Green vegetables	More than 4 days/week, 3-4 days/week, 1-2 days/week, occasionally
Number of meals per day	1, 2, or 3
Go to bed hungry	Sometimes, never
Go to bed bloated	Sometimes, never
General health	Very good, moderate, ok, poor
Malaria recently (3 months)	Yes, no
Stressed	Yes (money, relationships, exams, accommodation, water supply, noise), no
Number of hours of sleep	4, 5, 6, 7, or 8
Family deaths (last 2 years)	None, heart failure, high blood pressure, diabetes, other

## Abbreviations

BP: Blood pressure
BP-S: Systolic blood pressure
BP-D: Diastolic blood pressure
BMI: Body mass index
D/W: Days per week
EVD: Ebola virus disease
HF: High fever
HBP: High blood pressure
LGV: Leaf green vegetables
NS: Not significant
PI: Principal investigator
SSA: Sub-Sahara Africa.
